# Bioavailability of Tea Polyphenols: A Key Factor in Understanding Their Mechanisms of Action In Vivo and Health Effects

**DOI:** 10.1021/acs.jafc.4c09205

**Published:** 2025-02-07

**Authors:** Mingchuan Yang, Xiangchun Zhang, Chung S. Yang

**Affiliations:** † 243823 Tea Research Institute, Chinese Academy of Agricultural Sciences, Hangzhou 310008, China; ‡ Department of Chemical Biology, Ernest Mario School of Pharmacy, Rutgers, The State University of New Jersey, Piscataway, New Jersey 08854-8020, United States

**Keywords:** tea polyphenols, bioavailability, mechanisms, health effects, gut microbiota, metabolism

## Abstract

Tea polyphenols (TPP) are key contributors to the beneficial health effects of green tea and black tea. However, their molecular mechanisms of action remain unclear. This article discusses the importance of the bioavailability of TPP in understanding their mechanisms of action and health effects of tea consumption. The systemic bioavailability is rather high for smaller catechins, low for galloyl catechins, and very low or null for oligomers and polymers from black tea. The bioavailability of TPP oxidation-derived polymers and self-assembled nanomaterials is not clearly known. If the large molecular weight TPP cannot get into systemic circulation, then the biological activities and mechanisms of action derived from studies in vitro are unlikely to be relevant to their actions in internal organs in vivo. In that case, their interactions with microbiota and actions on the epithelial cells of the gastrointestinal tract are important to their health effects. Therefore, the bioavailability of different types of TPP is an important factor in determining their mechanisms of action and the health effects of tea consumption.

## Graphical abstract

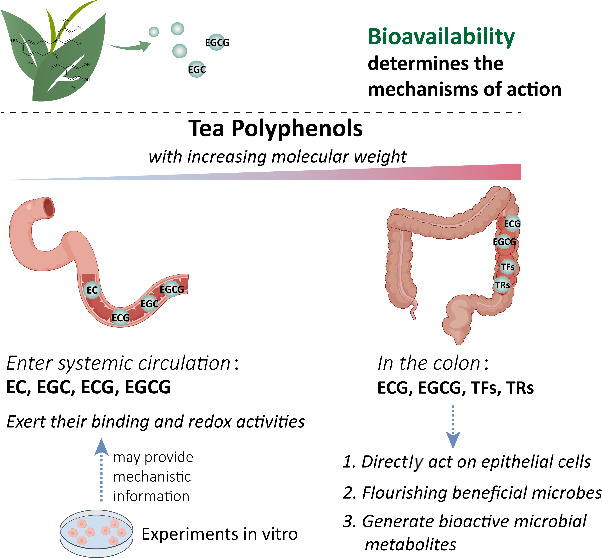

## 1 Introduction

Tea, made from leaves of the plant *Camellia sinensis*, is one of the most widely consumed beverages worldwide. Tea leaves are rich in polyphenols, of which 60–80% are catechins. The most consumed teas are green tea and black tea, accounting for 20% and 78% of world tea production, respectively. In green tea, catechins with varied bioavailability are the major active constitutions.^1^ In the manufacturing of black tea, the catechins are mostly converted to dimeric theaflavins (TFs) and polymeric thearubigins (TRs). These large molecular weight tea polyphenols (TPP) have very low or null systemic bioavailability in animals and humans.^2,3^ Other known active constituents in tea are caffeine and the unique amino acid theanine.

Tea consumption has been reported to have various beneficial health effects in humans. For example, the consumption of tea is associated with the reduction of cardiovascular disease mortality in large cohort studies, lower risk for certain types of cancers, lower incidence of type 2 diabetes in most of the studies, and higher cognitive functions in seniors in some studies.^4–7^ Recent studies also demonstrated an anti-inflammatory effect,^8^ uric acid lowering effect,^9^ and protective effect against aging-related muscle loss.^10^ These beneficial effects have been attributed to TPP, although caffeine and theanine may also contribute to the beneficial effects.

Many of these healthy effects have been demonstrated with TPP in human trials as well as in many studies in animal models.^4–10^ Numerous related studies have also been conducted in vitro, yielding data on the alteration of various signaling molecules by tea catechins, and many related mechanisms of action have been proposed.^11–13^ However, the lack of consideration of the bioavailability of the large molecular weight polyphenols by some authors caused confusion in many of the proposed mechanisms of action as well as unrealistic expectations for the health effects of tea. For large molecular weight polyphenols that cannot be absorbed, their actions on the epithelial cells of the gastrointestinal tract and actions mediated by gut microbiota should play important roles in the beneficial effects on health. Recent studies in the interactions between TPP and intestinal microbiota increased our understanding of the biological activities of TPP.^14^ However, most of these are correlational studies, and the involvement of other mechanisms cannot be excluded.

The most abundant catechin, (−)-epigallocatechin gallate (EGCG), and other polyphenols are readily oxidized in solution at neutral or alkaline pH to form large molecular weight polymers.^15^ These large weight aggregates were previously considered biologically inactive or irrelevant. However, some recent studies have demonstrated interesting biological activities of EGCG-autoxidation derived polymers.^16,17^ These novel laboratory-produced polymers will be discussed together with TRs, TFs, and catechins on their systemic bioavailability, actions after administration, interactions with microbiota in the gastrointestinal tract, and possible health effects. We hope that this review will facilitate better understanding of the mechanisms of action of different TPP as well as the beneficial health effects of tea.

## 2 Catechins in Green Tea

Green tea is manufactured through withering, drying, and roasting the tea leaves. The major tea catechins are EGCG, (−)-epigallocatechin (EGC), (−)-epicatechin-3-gallate (ECG), and (−)-epicatechin (EC). The structures of these catechins are shown in Figure 1. A typical brewed green tea beverage (e.g., 2.5 g tea leaves in 250 mL of hot water) contains 240–320 mg of catechins, of which 60–65% is EGCG, and 20–50 mg of caffeine.^1,18^

**Figure 1:**
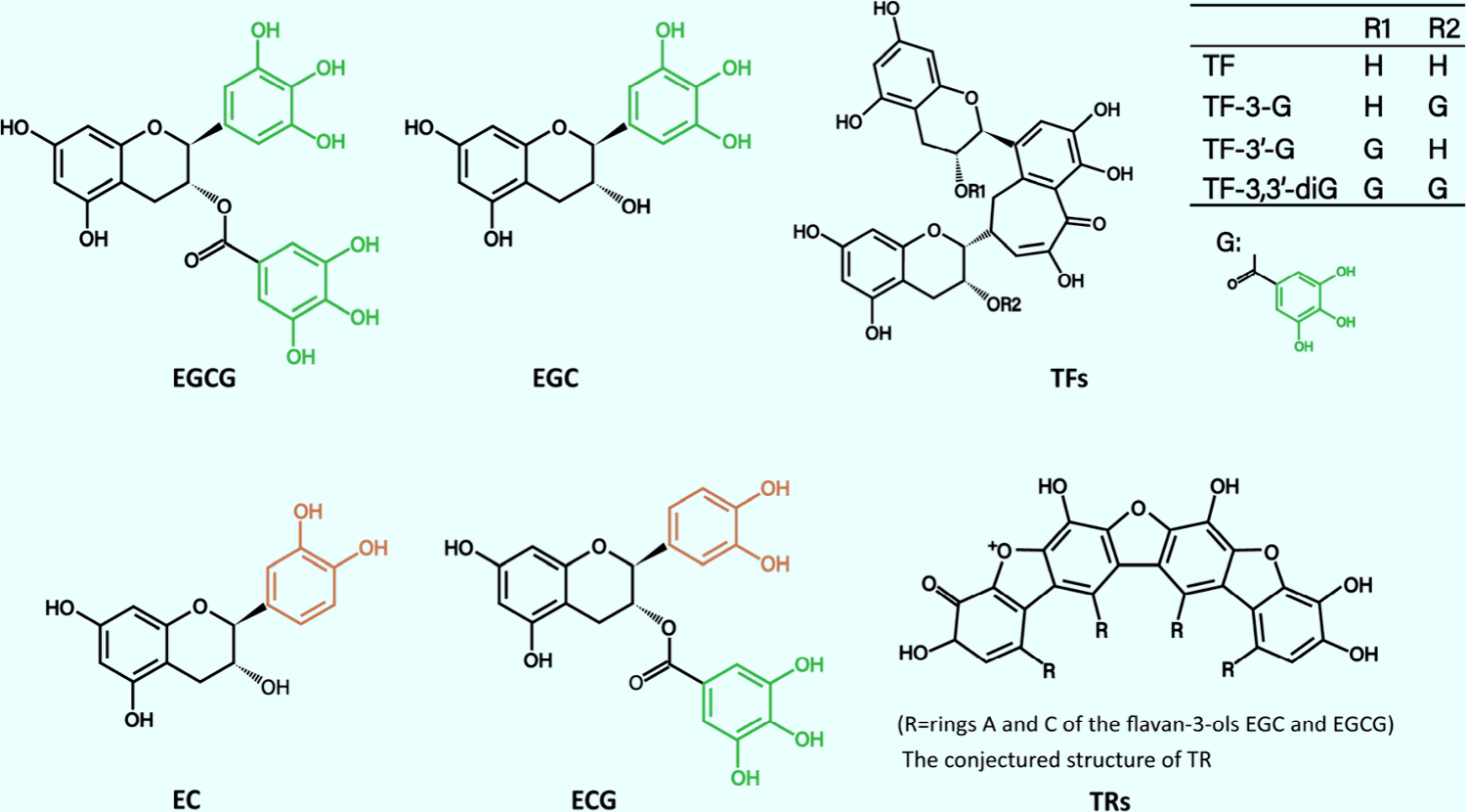
Structures of (−)-epigallocatechin-3-gallate (EGCG), (−)-epigallocatechin (EGC), (−)-epicatechin (EC), (−)-epicatechin-3-gallate (ECG), theaflavins (TFs), and thearubigins (TRs), conjectured from Haslam, E. Phytochemistry 2003, 64, 61–73, 10.1016/s0031-9422­(03)­00355-8 12946406 .

### 2.1 Absorption and Bioavailability of Tea Catechins

The absorption of orally administered TPP is dependent on the molecular size and the number of phenolic groups.^1,19^ In addition, a large portion of the absorbed EGCG and ECG is exported by multidrug-resistant proteins and other proteins to the ileum. These catechins can be reabsorbed and undergo enterohepatic circulation. The EGCG and ECG in the colon are metabolized by the microbes or excreted in the feces. Only trace amounts of EGCG are excreted in the urine. On the other hand, most of the absorbed EGC and EC are not exported back to the intestine, showing much higher systemic bioavailability, and excreted in the urine. Studies in both humans and animal models have shown that the oral bioavailability of EC (290 Da, 5 phenolic groups) is much higher than that of EGCG (458 Da, 8 phenolic groups), and there is species variation. The bioavailability of catechins generally follows the ranking order of EGCG < ECG < EGC < EC. In rats, following intragastric (i.g.) administration of decaffeinated green tea (200 mg/kg), the plasma bioavailability of EGCG, EGC and EC was 0.1, 14, and 31%, respectively. However, the plasma bioavailability of administered of EGCG in mice was much higher, with most of EGCG present as glucuronide conjugates. In humans, following oral administration of the equivalent of two or three cups of green tea, the peak plasma levels of EGCG (including the conjugated forms) were usually 0.2–0.3 μM. With high pharmacological oral doses of EGCG, peak plasma concentrations of 2–9 μM and 7.5 μM were reported in mice and humans, respectively.^1,20^

Several investigators have reported the pharmacokinetics of TPP in human volunteers.^20–22^ For example, after oral administration of 20 mg green tea extract per kg body weight, it took 1.4–1.6 h for the catechins to reach peak plasma concentrations.^20^ The maximal plasma concentrations for EGCG, EGC, and EC were 0.17, 0.73, and 1.41 μM, respectively, with corresponding terminal half-lives of 3.4, 1.7, and 2.0 h. Plasma EGC and EC were present mainly in the conjugated forms, whereas 77% of EGCG was in the free form. Methylated EGCG and EGC were also present in human plasma.^1,20^ Chow et al.^22^ demonstrated that the oral availability of EGCG was decreased by the presence of food and increased by continuous oral administration of EGCG (800 mg, once daily for 4 weeks); the molecular basis for this observation remains to be investigated.

### 2.2 Biological Activities of Tea Catechins

Tea and tea catechins have been shown or have been suggested to have inhibitory activities against cancer, obesity, cardiometabolic diseases, neurodegeneration, inflammation, and other diseases.^4,5,15,23,24^ The activities have been attributed mainly to EGCG. Similar but lower activities have also been shown for other catechins, generally following the ranking order of EGCG > ECG > EGC > EC. Concerning the mechanisms of actions of tea catechins, most studies have focused on their direct binding and redox activities. As reviewed previously,^15^ the redox and binding activities of tea catechins have been studied extensively using EGCG as an example. In recent years, only the interactions of tea catechins with microbiota in the gastrointestinal tract have received due attention. These activities are discussed below.

#### 2.2.1 Redox Activities of Tea Catechins

EGCG has direct antioxidant activities to affect redox-sensitive proteins. It can also be oxidized to produce oxidative stress and EGCG quinone, leading to the activation of Nrf2-regulated cytoprotective enzymes, including antioxidative enzymes, and thus, this can be considered as indirect antioxidant activity. These activities have been demonstrated in cell lines, animal models, and humans.^15^ The indirect antioxidant activity of EGCG may be more important than the direct antioxidant activity in beneficial health effects. On the other hand, consumption of excessive amounts of EGCG has been shown to cause liver toxicity.^5^

#### 2.2.2 Binding Activities of Catechins

High affinity binding of catechins can be measured using physical methods. For example, with NMR spectroscopy, EGCG was shown to bind to the BH3 pocket of antiapoptotic Bcl2 proteins, with an inhibition constant (*K*_i_) of 0.33–0.49 μM.^23^ Studies with surface plasmon resonance (SPR) found that EGCG (and ECG) could bind tightly to the signal transduction activator of transcription 1 with a *K*_d_ of 23 nM in MDA-MB-231 breast cancer cells.^24^ Binding of EGCG to the cell surface 67 kDa Laminin receptor (67LR) (with a *K*_d_ value of 0.04 μM) was first observed by Tachibana’s group using an SPR assay.^25^ Many subsequent studies by this group and others have led to the proposal that 67LR is the target for EGCG not only in its anticancer but also in its anti-inflammatory, antiallergic, and antiobesity effects as reviewed previously.^15,26^ More recent work by the Tachibana group suggested that EGCG underwent oligomer formation after binding to 67LR on the cell surface.^27^ The chemical nature of the suggested EGCG oligomerization and the functional role of this process remain to be investigated. Most of the above studies were conducted in cell culture systems; it is unclear whether the proposed targets for EGCG apply to the situation in vivo. With many other mechanisms that have been proposed for the action of EGCG in the literature, 67LR cannot be considered as a receptor for EGCG that mediates all of the biological activities of EGCG.

EGCG to enzymes can be assessed by the inhibition of enzyme activities. EGCG has been shown to inhibit the activities of a variety of enzymes,^15^ such as dihydrofolate reductase, glucose-6-phosphatedehydrogenase, and DNA methyl transferase I. Possibly due to high affinity binding to proteins, the IC_50_ values are usually higher when more protein is present in the assay mixture and even higher when the activities are measured in cell lysates. Interestingly, the inhibition of glyceraldehyde-3-phosphate involves the covalent binding of EGCG quinone to the active-center cysteine of the enzyme, suggesting the binding is preceded by the redox action of EGCG.^28^ In many of the studies described above, the nature of the inhibition has not been characterized fully, and the relevance of these observations in vivo remains to be evaluated.

#### 2.2.3 Binding and Redox Activities of Catechins in the Intestinal Tract

Tea catechins have been reported to inhibit activities of amylase, proteinases, and lipases, as well as physical binding to lipids and proteins, to decrease the digestion and absorption of carbohydrates, proteins, and lipids.^29–33^ As a consequence, increased fecal excretion of lipids and total nitrogen has been reported.^29,34^ These actions could decrease the total caloric intake, reduce body weight gain, and decrease the risk for metabolic diseases, especially in individuals taking a high fat, high caloric diet. Tea catechins may also influence gastrointestinal epithelial cells through direct contact as have been proposed for the prevention of oral and colon cancer, even by TPP that are not systemically available.^35,36^ However, this possibility remains to be further investigated.

As will be discussed below, the antioxidant activity of TPP has been proposed to provide a more anaerobic condition in the colon that helps to alter the composition of the gut microbiota, favoring the growth of beneficial microbes and contributing to the health beneficially effects.

#### 2.2.4 Degradations of Catechins by Colonic Microbiota

Catechins are extensively degraded by microbes in the intestine.^37–39^ The microbial enzymes catalyze the hydrolysis of the ester bonds of EGCG and ECG to produce gallic acid, as well as the fission of the C-ring of catechins to produce metabolites: 5-(3′,4′,5′-trihydroxyphenyl)-γ-valerolactone, 5-(3′,4′-dihydroxyphenyl)-γ-valerolactone, and 5-(3′,5′-dihydroxyphenyl)-γ-valerolactone.^38,40^ These metabolites are further degraded to phenylvaleric acid, phenolic acid, and smaller molecules.^41,42^ These C-ring fission metabolites and gallic acid can be absorbed to enter circulation, exert their biological activities, and be excreted in the urine.^14^ The redox and other biological activities of these C-ring metabolites are weaker than EGCG in vitro.^43^ To the best of our knowledge, there is no publication on their bioactivities in vivo. Our assessment is that these C-ring fission metabolites do not play a significant role in the biological activities of catechins in vivo. However, the metabolite gallate has been shown to have beneficial healthy effects in animals.^44^

#### 2.2.5 Alterations of Intestinal Microbial by Tea Catechins

The antioxidant activities of tea catechins could make the gut environment more anaerobic, which promotes the growth of strictly anaerobic bacteria and species that can degrade catechins (including those producing butyrate), while suppressing certain facultative aerobic bacteria-including many opportunistic pathogens.^14^ The butyrate generated by the flourishing butyrogenic bacteria can serve as an energy source for colonocytes and promote their differentiation. The flourishing bacterial species also help to maintain an intact intestinal barrier and improve immune functions. These activities contribute to the health effects of tea, such as reduction of body weight gain and alleviation of diabetes as reviewed previously.^14^ More recent studies showed that the interactions between EGCG and gut microbiota are also involved in the amelioration of hyperlipidemia,^45^ nonalcoholic liver disease,^46^ obesity-exacerbated lung cancer progression,^47^ microplastic-induced liver injuries,^48^ and even in the hypouricemic of EGCG in rodent models.^49^ Most of these publications are based on correlational studies; the involvement of other mechanisms of action cannot be excluded.

### 2.3 Importance of Bioavailability in Understanding the Mechanisms of Action and Health Effects of Tea Catechins

If a compound is not systemically bioavailable, then the binding and redox activities described above would not occur in internal organs in vivo. Even if the compound is bioavailable, the proposed mechanisms based on cell line studies may not be relevant in vivo, because of the differences of the concentrations of catechins used, the cell types used, and the cell environments; e.g., the oxygen partial pressure of the cell culture system is much higher, and the cultured cells are under much higher oxidative stress than those in vivo.^15^ That is why many of the activities of EGCG observed in cultured cells could not be observed in animals in vivo. Thus, many of the proposed health effects of EGCG or green tea based on observations in vitro without considering bioavailability should be viewed critically. For example, numerous anticancer mechanisms of EGCG have been proposed;^11,26,50^ however, the only known approved drug is Veregen—a green tea polyphenol preparation for topical application to treat genital warts.^51^

Orally administered TPP may affect the epithelial cells in the gastrointestinal tract by direct contact, which could be an important mechanism of action for molecules with low bioavailability. EGCG has low availability, and most of it enters the colon to alter the composition of microbiota and to be degraded by microbiota to produce bioavailable and bioactive metabolites. This EGCG-microbiota interaction is expected to combine with the binding and redox activities of EGCG to contribute to the health effects of EGCG and green tea.

## 3 EGCG Oxidation Derived and Self-Assembled Polymers

### 3.1 EGCG-Autooxidation Derived Polymers (EAOP)

Due to the presence of multiple phenolic hydroxyl groups, TPP are unstable and prone to autoxidation in solution at neutral and especially alkaline pH. This oxidation process results in the formation of larger molecular aggregates (molecular weight >10 kDa) known as EAOP. Interestingly, these polymers have been reported to exhibit biological activities in different experimental systems, including antihyperglycemic effects in mice and anticancer activities in vitro.^16,17^ Wu et al.^16^ found EAOP coated on intraperitoneal tissues and organs such as liver, kidney, and pancreas after i.p. injection to db/db mice, leading to the regulation of the renin–angiotensin system (RAS) and alleviation of diabetic symptoms. A key question is how the EAOP, presumably unable to enter systemic circulation and into cells due to the large molecular weights, regulate the RAS in the liver, kidney, and pancreas. Yang et al.^17^ proposed that the EAOP bind onto the cell surface and regulate multiple RAS components by reacting with the sulfhydryl groups on the ectodomains of transmembrane proteins. It is unknown whether EAOP also affects other signaling pathways of the cells. The bioavailability issue of the EAOP, however, remains puzzling.

### 3.2 EGCG Assembled into Nanoparticles

Recent research has shown that during EGCG autoxidation, EAOP nanoparticles with particle sizes of 60–200 nm are formed, accounting for 37–56% of the oxidation products.^52^ With EGCG, the eight hydroxyl groups (forming hydrogen bonds) and three benzene rings (hydrophobic interactions) make it easily undergo autoxidation and self-assemble into nanoparticles. These nanoparticles were isolated from the oxidation products by centrifugation with a molecular weight cutoff device, and their size was determined by scanning electron microscopy. These nanoparticle-containing EAOP preparation demonstrated antioxidant activity in vitro and regulated hepatic redox homeostasis by activating Nrf2-dependent antioxidant enzymes when administered i.p. to mice.^52^ The mechanisms of action remain to be carefully investigated. This nanoparticle-containing EAOP preparation also contained free EGCG (>7%), which can contribute to some of the activities observed. The most interesting possibility is that the EGCG nanoparticles can enter the liver. However, this remains to be demonstrated.

Under the acidic conditions in the stomach, the oxidation and polymerization process cannot be reversed to generate the tea polyphenol prototype.^52^ Nevertheless, the polyphenol nanoparticle structure disintegrated under acidic conditions, with less than 20% of the original nanoparticles remaining after 5 h as reported by Ju et al.^53^ Therefore, the majority of the nanoparticles could not reach the intestine. Whether orally administered nanoparticles or their polyphenol prototype can enter systemic circulation to reach different organs must be studied in animal models.

Nanotechnology has been used to improve the cellular permeability of large molecules that cannot directly penetrate the cell membrane.^54^ These nanoparticles are actively incorporated into cells via different endocytic pathways, and the agents are released under the action of various lysosomal hydrolases.^55–57^ Nanotechnology-facilitated drug delivery systems have enabled the intracellular delivery of EGCG to increase its bioavailability and biological activity. For example, oral administration of EGCG-β-lactoglobulin complex to rats doubled the plasma concentration of EGCG, and the complex was more effective in the prevention of metabolic syndrome in mice fed a high-fat diet, as compared to free EGCG.^58,59^ EGCG encapsulated into nanocomplexes, assembled from caseinophosphopeptides and chitosan, significantly enhanced the permeability of EGCG across a Caco-2 cell monolayer.^60^ EGCG chitosan-triphosphate nanoparticles significantly enhanced intestinal absorption of EGCG in excised mouse jejunum in Ussing chambers.^61^

Hu et al.^62^ used amyloid fibrils to support the self-assembly of EGCG into hybrid nanofilaments and macroscopic hydrogels. After oral administration, the amyloid-EGCG hydrogels retained their bulk features in the stomach, small intestine, and then colon in 4 h in mice. The hydrogels altered the colon microbial community, particularly decreasing the family *Enterobacteriaceae*, and ameliorated colitis. The EGCG hydrogel was more active than free EGCG in ameliorating colitis. It is unclear whether the hydrogel structure decreased the systemic bioavailability of EGCG. It is also unclear whether direct contact of EGCG, in hydrogel or free form, with colonic epithelial cells played a role in the observed beneficial effects.

Natural products are increasingly recognized for their structural diversity and interactions with biological targets.^63^ Self-assembled EGCG nanoparticles or encapsulated EGCG with nanocarriers may contribute to the oral bioavailability of EGCG. Future research is needed to characterize the structure and stability of these nanoparticles, elucidate the mechanisms of self-assembly, and understand whether or how these nanoparticles enter the systemic circulation and into organs and cells.

### 3.3 Self-Assembly of Nanoparticles in Tea Infusions

The rich content of amphiphilic molecules in tea infusion may facilitate the self-assembly of nanoparticles, with diameters in the range of 180–400 nm in the infusions of green tea, black tea, or white tea.^64–67^ The nanoparticles were isolated by ultracentrifugation, and the size was determined by transmission electron microscopy. These tea nanoparticles contained polyphenols, proteins, polysaccharides, and/or caffeine. Our unpublished results indicate that nanoparticles with diameters ranging from 100 to 600 nm are present in the infusions of the six types of tea (green, white, yellow, oolong tea, black, and dark teas) examined. Under alkaline conditions, these nanoparticles had diameters ranging from 80 to 220 nm.

## 4 Polymeric Polyphenols in Black Tea

Black tea is produced through withering, rolling, fermentation, and drying. During fermentation, most of catechins are oxidized and polymerized under the catalysis of endogenous polyphenol oxidase, peroxidase, and other enzymes to form TFs, TRs, and even higher molecular weight polyphenols, e.g., theabrownins (TBs).^68,69^ The major TFs are TF (MW 564 Da), TF-3-gallate (TF-3-G, MW 704 Da), TF-3′-gallate (TF-3′-G, MW 704 Da), and TF-3,3′-digallate (TF-3,3′-diG, MW 868 Da), while TRs or TBs are a group of poorly characterized large molecular weight polymers (MW range 2–40 or 3.5–100 kDa, respectively).^70,71^ The structures of some of the black tea polyphenols are shown in Figure 1. In black tea, catechins, TFs, and TRs each account for 3–10%, 1–6%, and 5–20% of the dry weight, respectively, depending on the tea variety and processing techniques.

### 4.1 Absorption and Bioavailability of Polymeric Polyphenols

Mulder et al.^2^ reported that after oral administration of 700 mg of TFs to volunteers, peak levels of TFs in plasma and urine, as determined by HPLC-MS, only reached 10 μg/L and 4.2 μg/L, respectively, after 2 h. The absorption of orally administered TFs in humans was only about 0.001%. The very low or null bioavailability of TFs is supported by the conclusion of the human feeding and in vitro fecal incubation studies by Pereira-Caro et al.^3^ that TFs were not absorbed in detectable amounts either in the upper or the lower intestinal tract. As reviewed by Li et al.,^72^ TFs were not detected after oral administration of TFs solution in one study but were determined in trace amounts after oral ministration of black tea in another study. However, TFs were measured in several internal organs after intravenous administration.^72^ TRs, with an even larger molecular size, are expected to have even less or null systemic bioavailability. However, the presence of other constituents in black tea infusion may increase the bioavailability of TFs, and this possibility needs to be assessed experimentally.

### 4.2 Activities of Polymeric Polyphenols

TFs and TRs have been shown to have inhibitory activities against cancer, obesity, hyperuricemia, cognitive dysfunction, muscle atrophy, and other diseases in animal models.^73–77^ But their mechanisms of action and possible health effects in humans are unclear. Given the very low bioavailability of TFs and TRs, their biological activity can be attributed to their interactions with intestinal microbiota or their direct binding and redox activities in the gastrointestinal tract. Some of the biological activities, possible health effects, and mechanisms of action of TFs and TRs are discussed below.

#### 4.2.1 Redox Activities of Polymeric Polyphenols

TFs have a high antioxidant capacity in vitro and can effectively eliminate peroxides and free radicals due to their phenylpropanoid ketone structure and phenolic hydroxyl groups providing protons.^78,79^ TFs can also increase the activity of antioxidant enzymes, such as superoxide dismutase, catalase, and GSH-peroxidase in HFD-fed mice, and protect against cholesterol-induced oxidative injuries in HUVEC cells.^80^ On the other hand, the pro-oxidative effect of TFs in the production of ROS in cell culture has been proposed to induce cancer cell apoptosis.^81,82^

#### 4.2.2 Binding Activities of Polymeric Polyphenols

TFs have strong protein-binding activity. In a study investigating the mechanisms of interaction between TF with human glycosylated and nonglycosylated serum albumins, TF was found to bind serum albumin at site II. This suggests that TF can be transported with human serum albumin as a carrier.^83^ TFs may help remove toxic amyloid deposits by binding to two regions of the amyloid-β peptide, amino acids 12–23 and 24–36, and promoting the assembly of Aβ and αS into nontoxic spherical aggregates.^84^ In summary, TFs exhibit high affinity for binding to a range of proteins and enzymes, which may be manifested in antibacterial activities, digestive inhibition, and prevention of amyloid-related diseases if TFs can reach the appropriate sites of action.

Miao et al.^85^ investigated the inhibitory effect of TFs on alpha-amylase activity in vitro and by computer simulations. The inhibitory activity was attributed to the interaction between the hydroxyl and galloyl groups of TFs and the active site of alpha-amylase through hydrogen bonding and π–π interactions. Molecular dynamics simulations indicated that the TFs also have an affinity for the lipid bilayer headgroups via hydrogen bonding.^86^ These activities are expected to reduce the digestion and absorption of macronutrients, as discussed below.

#### 4.2.3 Direct Actions of TFs and TRs in the Gastrointestinal Tract

Similar to EGCG, TFs and TRs may affect gastrointestinal epithelial cells through direct contact and exert their binding and redox effects. Oral administration of TRs-like polymeric polyphenols from black tea inhibited carcinogen-induced colorectal tumorigenesis in rats and oral cancer in hamsters,^75,87^ but did not prevent benzo­[a]­pyrene-induced lung tumors in A/J mice.^88^ However, topical application of these polymers inhibited carcinogen-induced skin carcinogenesis in mice.^89^ These studies suggest the importance of direct contact of these polymers with the target cells in their disease preventive activities. It has been proposed that polyphenols may induce gastrointestinal hormone release through binding to extra oral taste 2 receptor interactions.^90^ This novelty mechanism remains to be substantiated experimentally. More importantly, through inhibiting the activities of alpha-amylase,^85^ alpha-glucosidase,^91^ and sucrase-isomaltase,^92^ and other digestive enzymes, as well as interfering the lipid micelle structure in the intestine, TFs may decrease the digestion and absorption of macronutrients, thus contributing to the weight reduction and hypoglycemic effects. We propose that TRs and larger molecular weight polyphenols have similar effects on the digestion and absorption of macronutrients in the gastrointestinal tract.

#### 4.2.4 Interactions between Polymeric Polyphenols and Intestinal Microbiota

The interactions between polymeric polyphenols and gut microbiota have been studied extensively. For example, Li et al.^76^ reported that TFs improved behavioral impairments through the microbiota-gut-brain axis in galactose-induced aging mice. TFs maintain gut homeostasis by increasing the relative abundances of *Actinobacteria* and the ratio of *Firmicutes* to *Bacteroidetes*, as well as decreasing the relative abundances of *Bacteroidetes* and *Proteobacteria*. Additionally, TFs prevented the galactose-induced reduction in the microbial production of short-chain fatty acids and essential amino acids. In vitro fecal microbial fermentation further demonstrated the TFs’ effects in enhancing *Bacteroides*, *Faecalibacterium*, *Parabacteroides*, and *Bifidobacterium*, while suppressing *Prevotella* and *Fusobacterium*.^93^ Henning et al.^94^ found that dietary black tea polyphenols decreased the abundance of cecum *Firmicutes* and increased the amount of *Bacteroidetes*, both of which were significantly correlated with weight loss in mice fed a high-fat diet. The hypolipidemic activity of TBs from Pu-erh tea was also found to be related to their regulation of gut microbiota in mice.^95^ TBs altered the gut microbiota in mice, predominantly suppressing microbes associated with bile-salt hydrolase activity. Germ-free mice receiving microbiota from TBs-fed mice showed lower weight gain, serum total cholesterol, and total triglyceride concentrations. These studies demonstrate that tea polyphenols exert a selective effect on gut microbiota, which can lead to beneficial health effects.

#### 4.2.5 Degradation of Polymeric Polyphenols by Colonic Microbiota

Gut microbiota play crucial roles in the biotransformation of TFs and TRs. Chen et al.^96^ found that the galloyl ester bond of TF-3,3′-diG was cleaved by microbial esterase to produce gallate in specific pathogen free mice, but not in germ free mice. In vitro microbial incubation using fecal slurries from three healthy individuals found that TF-3-G and TF-3′-G were cleaved to TF and gallic acid. Human oral administration and gut microbiota incubation studies have demonstrated the further degradation of TFs to 3-(4′-hydroxyphenyl) propionic acid and other ring fusion metabolites, together with gallic acid, methylated gallate, and various small molecules.^3^ This is similar to the microbial metabolism of tea catechins.^14^ These microbial metabolites may contribute to the biological activities of TFs. For example, 3-(4′-hydroxyphenyl)-propionic acid and gallic acid have been reported to protect neuronal cells from oxidative stress.^97^ As a microbial metabolite in mice, gallic acid may contribute to the uric acid lowering effect of TFs.^44^ Methylated gallic acids have been reported to induce apoptosis of human colon cancer cells^98^ and to reduce the serum levels of inflammatory mediators in endotoxemic mice.^99^

Similarly, after oral administration of a partially purified TRs fraction to mice, phenyl-γ-valerolactone and related ring-fission products were observed.^100^ These metabolites could enter systemic circulation and be excreted through urine. Phenyl-γ-valerolactones and their ring fission products have been reported to exhibit anti-inflammatory, antihypertensive, and anticancer activities in laboratory studies.^101^

### 4.3 Biological Effects of TPP in Black Tea and Mechanism Involved

As described above, if a compound is not systemically bioavailable, its biological activities observed in internal organs in vivo are unlikely to be the same as those observed in vitro. This concept can be applied to TFs and TRs, which have a very low or null systemic bioavailability. However, TFs and TRs have strong protein binding and redox activities. These activities could decrease the digestion and absorption of macronutrients as well as directly influence the epithelial cells in the gastrointestinal tract. However, the biological activities of TFs and TRs may mainly come from their interactions with gut microbiota and the activities of their metabolites, as shown in Figure 2. Therefore, the biological effects of black tea consumption can be due to the combined mechanisms of actions of the absorbed TPP and the TPP that are in the gastrointestinal tract as well as the actions of caffeine and theanine. The relative contribution of each of these actions depends on the different types of beneficial effects.

**Figure 2:**
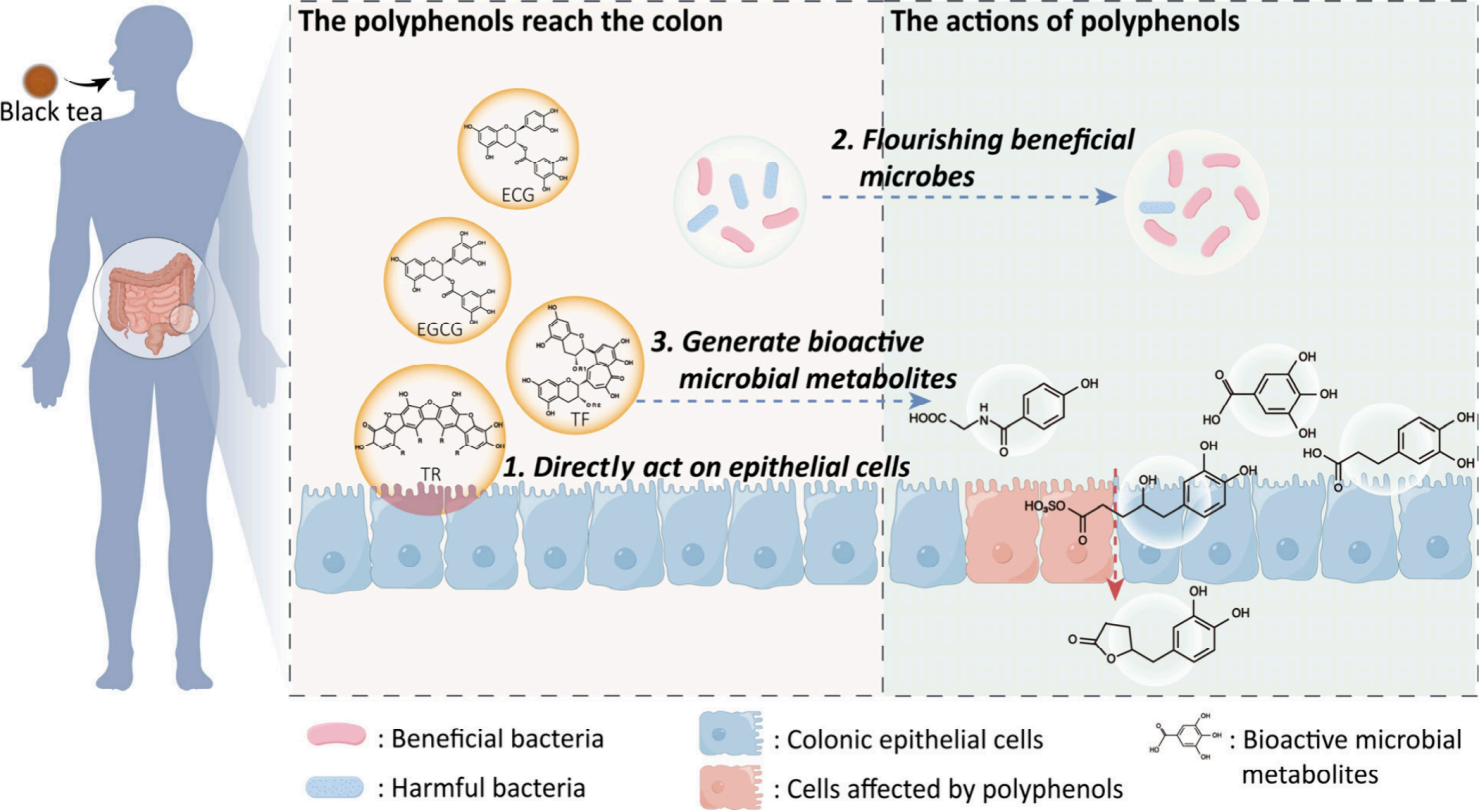
Actions of tea polyphenols (ECG, EGCG, TFs, and TRs) in the colon. 1). The polymeric polyphenols exert their binding and redox actions on the epithelial cells through direct contact. 2). These polyphenols can modify the intestinal microbiota in favor the growth of beneficial microorganisms to generate beneficial health effects. 3). Some microbial metabolites generated from the polyphenols have biological activities in vivo.

## 5 Bioavailability Determines the Mechanisms of Actions in Vivo

As shown in Figure 3, the bioavailability of different TPPs determines their mechanisms of action in vivo. Tea catechins can be absorbed in the gastrointestinal tract, enter systemic circulation, and reach many internal organs to exert their biological effects. Because of their molecular size and the number of phenolic groups, EC and EGC have reasonably good systemic bioavailability, while ECG and EGCG have rather low bioavailability, but the larger molecular weight TFs and TRs from black tea have very low or null availability. For orally ingested TPP that are not absorbed, their biological activities in vivo can be due to their direct action on the epithelial cells of the gastrointestinal tract or their interactions with gut microbiota. Therefore, the mechanisms of action proposed for these larger TPP based on studies in cell lines may not be relevant to the biological activities observed in the internal organs of animals

**Figure 3:**
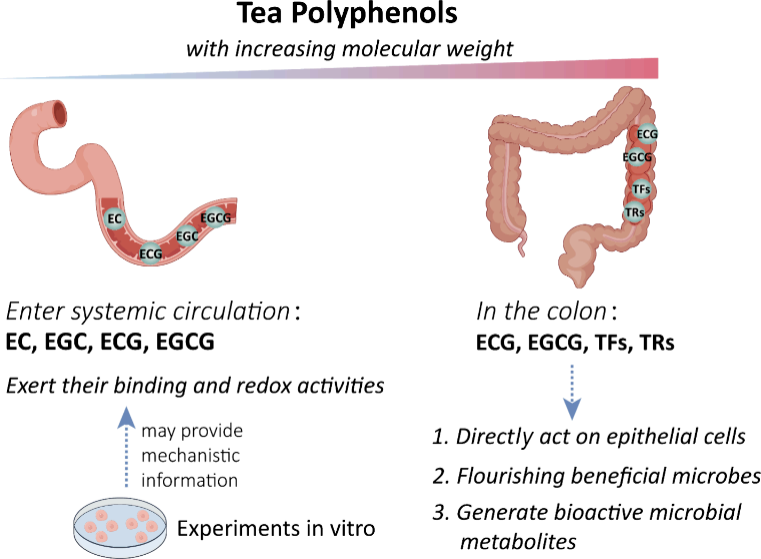
Bioavailability determines the mechanisms of action in vivo. After oral administration of tea polyphenols, catechins are absorbed in the small intestine and enter systemic circulation to reach many organs and tissues to exert their binding and redox activities; in vitro experiments may provide useful information on the mechanisms of action. The larger molecular weight polyphenols enter the colon may exert their binding and redox actions on the epithelial cells through direct contact. More importantly, these polyphenols can influence the intestinal microbiota in favor of the growth of beneficial microorganisms to generate beneficial health effects. Some microbial metabolites generated from polyphenols may also have biological activities in vivo.

In addition to the bioavailability issue, there are many differences between cells in the culture media and cells in vivo. The former are exposed to much higher oxygen partial pressure than the cells in vivo,^102^ and the culture cells are under high oxidative stress because of the prooxidant activity of EGCG or other catechins—high levels of hydrogen peroxide are produced, and the cellular level of glutathione is decreased. The cultured cell lines are different in cell physiology from the normal cells in animals. Even if the beneficial health effects can be demonstrated in animals, such effects cannot be extrapolated to humans without reliable human studies because of the species difference as well as the differences in the dose and other conditions used for the studies. We hope this concept will help to clarify some of the confusion in the literature and avoid wasting energy in testing unworthy hypotheses or developing tea-based dietary supplements that cannot produce the claimed beneficial effects.

## Abbreviations

TPP: tea polyphenols
TFs: theaflavins
TRs: thearubigins
TBs: theabrownins
EGCG: (−)-epigallocatechin gallate
EAOP: EGCG-autoxidation derived polymers
EGC: (−)-epigallocatechin
ECG: (−)-epicatechin-3-gallate
EC: (−)-epicatechin
SPR: surface plasmon resonance
STAT1: signal transduction activator of transcription 1
67LR: 67 kDa Laminin receptor
RAS: renin–angiotensin system
TF-3-G: TF-3-gallate
TF-3′-G: TF-3′-gallate
TF-3,3′- diG: TF-3,3′-digallate.
